# A Scoping Review of Breastfeeding Interventions and Programs Conducted Across Spanish-Speaking Countries

**DOI:** 10.1177/15248399241237950

**Published:** 2024-03-25

**Authors:** Silvana Blanco, Basil H. Aboul-Enein, Nada Benajiba, Elizabeth Dodge

**Affiliations:** 1 Northeastern University, Boston, MA, USA; 2 London School of Hygiene & Tropical Medicine, London, UK; 3 Ibn Tofail University-CNESTEN, Kenitra, Morocco; 4 University of New England, Portland, ME, USA

**Keywords:** breastfeeding, lactation, maternal-child health, Spanish-speaking countries

## Abstract

Breastfeeding is vital to a child’s lifelong health and has significant positive benefits to mother’s health. World Health Organization recommends beginning exclusively breastfeeding within the first hour after birth and continuing during the first 6 months of infant’s life. The purpose of this review is to identify and examine breastfeeding interventions conducted across the Spanish-speaking countries. A scoping review of the literature was conducted across 14 databases for relevant publications published through April 2023 to find studies in Spanish-speaking countries that involved breastfeeding as an intervention component. A total of 46 peer-reviewed articles were included in this review, across 12 Spanish-speaking countries. Participants ranged from pregnant women, mothers, mother-infant pair, and health care professionals. Intervention at the individual level in combination with support from trained health care professionals or peer counselors seemed to have higher improvements in breastfeeding rates. The greatest improvement in exclusively breastfeeding for 6 months was seen in interventions that included prenatal and postnatal intensive lactation education, for a period of 12 months. The most effective interventions that improved rates of any breastfeeding included promotional activities, educations workshop, and training of health care staff along with changes in hospital care. Breastfeeding promotion is an economical and effective intervention to increase breastfeeding behavior and thereby improving breastfeeding adherence across Spanish-speaking countries.

While health organizations and medical experts worldwide agree on the importance of breastfeeding, there have been poor improvements in the practice of breastfeeding worldwide (Cai et al., 2012; Walters et al., 2019). According to the World Bank’s most recent statistics, in Latin America and the Caribbean, the rate at which infants less than 6 months of age that are exclusively breastfed is at 37% (Arocha-Zuluaga et al., 2022). Some countries in Latin America and the Caribbean present with alarmingly suboptimal rates, including Dominican Republic, 5%; Venezuela, 7%; and Panama, 21% (Arocha-Zuluaga et al., 2022). These rates fall very short of the World Health Organization’s (WHO’s) 2025 goal of increasing the rate of exclusively breastfeeding in the first 6 months of life to at least 50% (Walters et al., 2019; World Health Organization, 2021). Reaching these goals will require action at the individual, community, and health institutional levels.

Furthermore, it is important to understand the barriers affecting mothers from breastfeeding. These barriers can be impacted by factors at the individual, community, and health institutional levels (Snyder et al., 2021). Causes such as low self-efficacy, lack of familial support (individual level), lack of cultural acceptance of breastfeeding (community level), or lack of counseling and education from health care personnel (health institutional level) can hinder a mother from breastfeeding or exclusively breastfeeding (Atyeo et al., 2017; Colombara et al., 2015; Snyder et al., 2021). For these reasons, it is important to develop culturally congruent interventions to promote, support, and encourage mothers to breastfeed and achieve the WHO 2025 goal of extending the duration of exclusive breastfeeding for up to 6 months of age to 50%.

In Spanish-speaking sovereign countries (SSSCs), such as in Latin America and the Caribbean, more than 50% of all infants are not breastfed within the first hour of life, 37% are breastfed exclusively for the first 6 months of life, and between 31% and 55% continue to receive breastmilk for up to 2 years of age (Caribbean Public Health Agency, 2022). Not meeting the WHO/United Nations International Children’s Emergency Fund (UNICEF) breastfeeding recommendations has been linked to child malnutrition (Atyeo et al., 2017). What is needed is a communal approach in the development and implementation of interventions that promote breastfeeding practices in SSSCs.

The aim of this review is to identify breastfeeding-promotion interventions across SSSCs and help guide researchers and health care professionals in summarizing the current state of the existing literature, identify gaps in the existing literature, and identify areas where future work is needed.

## Methods

### Literature Search

A scoping review of the literature was conducted using 14 databases (Table 1), employing the PRISMA Extension for Scoping Reviews (Tricco et al., 2018), and the same search strategy was utilized in each of the 14 databases, using all the keywords, search terms, and phrases included in Table 1. This search was done in collaboration with a university librarian. For the purpose of this review, SSSC included Argentina, Bolivia, Costa Rica, Chile, Colombia, Cuba, Dominican Republic, Ecuador, El Salvador, Equatorial Guinea, Guatemala, Honduras, Mexico, Nicaragua, Panama, Paraguay, Peru, Spain, Uruguay, and Venezuela. In addition, reference lists of relevant studies were screened to identify publications from other studies that might be eligible for this review.

**Table 1:** Electronic Databases Used With Relevant Search Period and Terms

***Databases***	***Search period***	***MeSH keywords, terms, phrases, and Boolean operators***
Article First; Biomed Central; BioOne; BIOSIS; CINAHL; EBSCOHost; ProQuest; PubMed; SAGE Reference Online; ScienceDirect; Scopus; SpringerLink; Taylor & Francis; and Wiley Online	Up to April 30, 2023	(All fields) Breastfeeding [MeSH Terms]) OR Lactation OR Lact^a^ [MeSH Terms]) OR Amamantamiento [MeSH Terms]) OR leche maternal [MeSH Terms]) OR Lactancia maternal [MeSH Terms]) OR Lactación [MeSH Terms]) AND (All fields) education OR educación OR educat^a^ [MeSH Terms]) OR promotion OR promoción OR promot^a^ [MeSH Terms]) OR intervention OR intervención [MeSH Terms]) OR Program^a^ [MeSH Terms]) AND (All fields) Argentina; OR Bolivia; OR Chile; OR Colombia; OR Costa Rica; OR Cuba; OR Dominican Republic; OR Ecuador; OR El Salvador; OR Equatorial Guinea; OR Guatemala; OR Honduras; OR Mexico; OR Nicaragua; OR Panama; OR Paraguay; OR Peru; OR Spain; OR Uruguay; OR Venezuela

a Based on the PRISMA Extension for Scoping Reviews (Tricco et al., 2018), the same search strategy was employed in each of the 14 databases listed, using all the keywords, search terms, and phrases included above.

### Eligibility Criteria

The inclusion criteria were set to intervention studies published up until April 30, 2023, in peer-reviewed journals. English and Spanish peer-reviewed intervention-based studies with quantitative outcomes were included. Inclusion criteria consisted of pregnant women/mothers across SSSCs receiving education, training, or other intervention that promotes breastfeeding or exclusive breastfeeding (Table 2). Only intervention-focused articles that involved the promotion of either any breastfeeding or exclusive breastfeeding for a defined period of time as either the primary intervention or as a component of a multi-behavioral intervention at all levels (individual, programmatic, community, family, or policy) were included. Studies that examined Spanish-speaking communities or migrants of Spanish origin residing in non-Spanish–speaking countries were excluded.

**Table 2:** PICOS Criteria for Inclusion and Exclusion of Studies

***Parameter***	***Inclusion criteria***	***Exclusion criteria***
**Population**	• Pregnant woman/mothers residing in Spanish-speaking sovereign countries • Healthcare providers • Health centers and hospitals	• Non-Spanish-speaking sovereign countries
**Intervention type**	Any type of education intervention that promotes BF or EBF, including: • Educational interventions • Training intervention • Multicomponential interventions • Intervention promoted any level of influence, i.e., individual, programmatic, community, family, or policy level	• Interventions that are not delivered in Spanish-speaking sovereign countries • Interventions that do not address BF nor EBF-related outcomes • Spanish-speaking diaspora
**Comparators**	Preintervention, baseline BF- and EBF-related variables (knowledge, attitudes, practice, implementation of BF-promoting programs) of studied groups who were: • Control: received no intervention. • Intervention: receive intervention(s) • Postintervention • Intervention follow-up	• N/A
**Outcomes of interest**	• Changes in knowledge • Changes in attitudes • Changes in practice • Change in BF rate • Change in EBF rate ○ In: Mothers and in health care providers, health center, and hospital practices, and BF/EBF at discharge	• Non-BF- and non-EBF-related outcomes
**Language**	English and/or Spanish	All other languages
**Study type**	Peer-reviewed original research articles Original research conference publications Experimental intervention studies with quantitative outcomes, at both population and community levels, as well as health care institutions	Non-peer-reviewed articles Commentaires Narratives Communications Non-intervention-based studies White papers Gray literature Qualitative studies Nonnumeric/categorical assessments or qualitative studies

*Note.* BF = breastfeeding; EBF = exclusive breastfeeding; N/A = not applicable.

### Study Selection and Data Extraction

**Figure 1:**
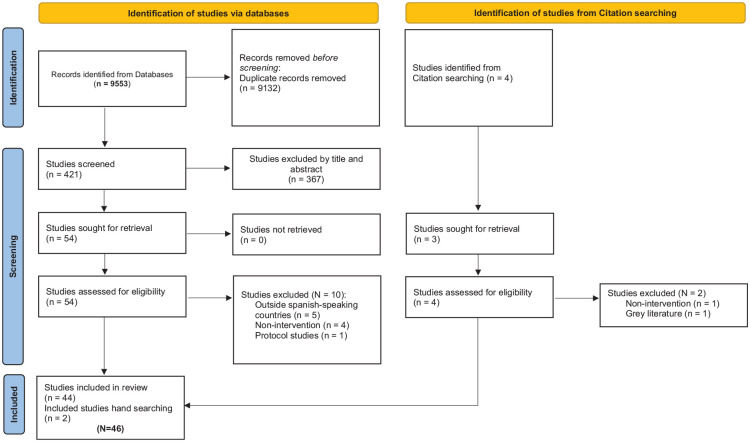
Flow Diagram

BAE and SB independently conducted the literature search and selected studies for inclusion in the scoping review. Differences were discussed to reach consensus; ED resolved discrepancies if needed. Extraction and tabulation of data were done by SB and independently checked by BHA-E and NB. The search strategy was adapted according to the indexing systems of each respective database. Rayyan QCRI software (Ouzzani et al., 2016; Rayyan, 2021) was used to assist in the screening process and study selection. Titles and abstracts were screened for relevancy, and potentially relevant journal abstracts were reviewed by three of the authors (NB, ED, and BAE). Potential studies for inclusion in this review were evaluated independently by each author for relevance, merit, and inclusion/exclusion criteria (Table 2). All selected articles were then discussed with the primary author before taking the final decision for inclusion (Figure 1). Once the list of selected studied was finalized, BAE extracted and SB and NB cross-checked the following for each study: author, date, target population, country, type of study, sample size, type, details of intervention, measured parameters, main results, and main recommendations. Differences in opinion in data extracted were discussed to reach consensus and tabulated. Given that methodological quality assessment is not a prerequisite for scoping reviews, we did not appraise the included studies (Peters et al., 2020).

## Results

In total, 46 peer-reviewed articles were included in this review, across 12 SSSCs: 16 from Spain (Artieta-Pinedo et al., 2013; Balaguer Martínez et al., 2018; Cebrián et al., 2006; Estalella et al., 2020; Franco-Antonio et al., 2020; Gámez Requena et al., 2004; Gonzalez-Darias et al., 2020; Guijarro et al., 2014; Hernández Perez et al., 2018; Lasarte Velillas et al., 2007; Martínez Galiano & Delgado Rodríguez, 2013; Martínez-Galiano & Delgado-Rodríguez, 2014; Roda et al., 2002; Santamaría-Martín et al., 2022; Seguranyes et al., 2014; Soriano-Vidal et al., 2018), 10 from Mexico (Bolaños-Villar et al., 2023; Bueno-Gutiérrez et al., 2021; Iñarritu-Pérez et al., 2022; Langer et al., 1998; Monterrosa et al., 2013; Morrow et al., 1999; Ortiz-Félix et al., 2021; Perez-Escamilla et al., 1993; Rodriguez-Garcia et al., 1990; Sanchez-Espino et al., 2019), seven from Chile (Alvarado et al., 1996; Burkhalter & Marin, 1991; Lucchini Raies et al., 2013; A. Pérez & Valdés, 1991; Pugin et al., 1996; Valdés et al., 1993, 1995), three from Peru (Chumpitaz Durand et al., 2021; Leyva et al., 2015; Velásquez Rondón & Huaman Hernández, 2020), two from Colombia (Arias Ramírez et al., 2021; Ulloa Sabogal et al., 2023), one from Honduras (Cohen et al., 1999), two from Cuba (Morales et al., 2021; Tamayo Velázquez et al., 2022), one from Dominican Republic (Navarro et al., 2013), one from Ecuador (Maslowsky et al., 2016), one from Nicaragua (K. Perez et al., 2018), one from Venezuela (Rojas et al., 2019), and one from Guatemala (Prieto et al., 2017; Table 3). Study designs included 14 randomized control trials (Balaguer Martínez et al., 2018; Bueno-Gutiérrez et al., 2021; Cohen et al., 1999; Franco-Antonio et al., 2022; Gonzalez-Darias et al., 2020; Hernández Perez et al., 2018; Langer et al., 1998; Lucchini Raies et al., 2013; Morrow et al., 1999; Perez-Escamilla et al., 1993; Rodriguez-Garcia et al., 1990; Santamaría-Martín et al., 2022; Seguranyes et al., 2014; Ulloa Sabogal et al., 2023), 14 quasi-experimental studies (Arias Ramírez et al., 2021; Burkhalter & Marin, 1991; Estalella et al., 2020; Iñarritu-Pérez et al., 2022; Lasarte Velillas et al., 2007; Monterrosa et al., 2013; Morales et al., 2021; Navarro et al., 2013; Ortiz-Félix et al., 2021; Perez-Escamilla et al., 1993; Pugin et al., 1996; Tamayo Velázquez et al., 2022; Valdes et al., 1995; Velásquez Rondón & Huaman Hernández, 2020), 12 prospective (Alvarado et al., 1996; Artieta-Pinedo et al., 2013; Bolaños-Villar et al., 2023; Cohen et al., 1999; Guijarro et al., 2014; Martínez Galiano & Delgado Rodríguez, 2013; Martínez-Galiano & Delgado-Rodríguez, 2014; Maslowsky et al., 2016; K. Perez et al., 2018; A. Pérez & Valdés, 1991; Soriano-Vidal et al., 2018; Valdés et al., 1993), and three retrospective studies (Cebrián et al., 2006; Chumpitaz Durand et al., 2021; Gámez Requena et al., 2004), one cross-sectional study using educational sessions (Rojas et al., 2019), one educational intervention (Sanchez-Espino et al., 2019), and one quantitative pre-experimental with pre-post evaluation study (Leyva et al., 2015). Sample size ranged from 35 to 1,400 pregnant women/mothers. Two studies were a multi-part Breastfeeding Promotion Program, which included training of health care personnel, education, and activities in the prenatal and outpatient clinics, as well an open lactation clinic (Rojas et al., 2019; Valdes et al., 1995). Fathers or extended families were not involved in any of the included studies. Most of the studies reported exclusive breastfeeding (*N* = 24), any form of breastfeeding was reported in 11 studies, and 11 studies reported knowledge and attitude toward breastfeeding. Two studies involved helping mothers achieve re-lactation (Atalah et al., 2004; Cohen et al., 1999).

**Table 3:** Summary of Literature Search (*N* = 46)

***Authors (year)***	***Target population/country***	***Type of study***	***Sample size***	***Type and details intervention***	***Theoretical framework***	***Measured parameters***	***Main results***	***Main recommendations***
**Primary outcome: Exclusive breastfeeding**								
***Individual level***								
**Maslowsky et al. (2016)**	Inpatient mothers after delivery at a public hospital Quito, Ecuador	Prospective evaluation study	*N* = 135 IG: *n* = 75 CG: *n* = 60	CG: usual discharge instructions IG: standard instructions plus telephone delivered educational session from trained RN 48 h of discharge and access to on-call RN during 30 days for medical advice, information, and support Follow-up at 3 months	Not reported	EBF rates and satisfaction of mothers with intervention	IG reported higher rates of EBF; 87% compared to 67% of CG at 3 months. Mothers expressed satisfaction with intervention for positively impacting infants’ health (99%)	Low resource phone-based program has shown effective at supporting BF in low-resource country
**Pugin et al. (1996)**	Pregnant women Santiago, Chile	Quasi-experimental, pre-post intervention group	*N* = 752 IG: *n* = 422 (IG subset): 59 postintervention received 6^th^ visit. CG: *n* = 330	IG: Prenatal BF Skills Group Education (PBFSGE): Five-session educational program, including BF, delivered through prenatal checkups. IG (subset): Additional 6th group session discussing various aspects of BF and sharing personal experiences. Follow-up after 6 months CG: no intervention	Not reported	EBF patterns and duration	67% of IG EBF at 6 months, compared to 32% in CG. 80% of IG (subset) were EBF at 6 months, compared to 65% that did not receive the additional 6th session	BF educational group programs improves EBF practices; hands-on skills reinforcement has been shown to be a significant additive in improving BF practices
**Burkhalter et al. (1991)**	Infants Lo Barnechea, Chile	Quasi-experimental	3 Samples: CG: 130 infants IG: 100 infants IG-2: 105 (to test sustainability of the program)	Prenatal component: 4 BF promotion and educational programs-lectures and presentation regarding motivation and education on BF and EBF Postnatal component: infants weighed monthly plus 8 home visits from clinic staff. (Mothers with problems or who wanted to start supplementary feeds were visited weekly) CG: before program was initiated	Not reported	Duration EBF at 6 months	At 1 month % of EBF: CG: 85% IG: 95% IG-2: 87% At 6 months % of EBF: CG: 30% IG: 74% IG-2: 61%	This intervention included motivational education, and most important to the program’s success was the positive efforts to help mothers return to EBF through home visits by clinical staff and peer group encouragement.
**Lucchini Raies et al. (2013)**	Postpartum women Santiago, Chile	Parallel 2-arm randomized controlled trial	N: 649 IG: *n* = 330 CG: *n* = 319	IG: comprehensive care—early initiation of BF was promoted, educational support immediately postpartum and home visit 48 h from discharge, promoting BF CG: traditional care	Not reported	Onset and duration of EBF at 8 weeks	EBF at 8 weeks 56% in IG compared to 48% in CG	An intervention that considers a comprehensive delivery care with support of early initiation of BF has positive effect on BF practices
**Bueno-Gutiérrez et al. (2021)**	Mothers with infants under 4 months of age Tijuana, Mexico	Randomized controlled trial	*N* = 80 CG: *n* = 40 IG: *n* = 40	IG: Interpersonal counseling, focusing on solving BF obstacles identified by mother CG: counseling on standard feeding information Follow-up at 2 months	Theory of planned behavior	EBF practices	IG: 89% EBF compared to 33% of CG at 2 months and increase in BF attitudes (*p* = .0001), self-efficacy (*p* = .046)	The strength of this study used counseling technique that addressed the need of mothers along with training healthcare professionals with knowledge and skills to clearly promote BF
**Valdes et al. (1993)**	Mother-child pairs Santiago, Chile	Prospective intervention study	IG: *n* = 422 CG: *n* = 313	IG: BF Promotion Program (BFPP): training of health team, activities in the prenatal outpatient clinic, and maternity ward, plus open lactation clinic CG: entered study prior to BF promotion program Follow-up at 6 months	Not reported	EBF and early BF	Average time from birth to initiating BF: IG: 2.8 hours CG: 6.7 hours EBF at 6 months IG: 66.8% CG: 31.6%	BFPP produced a positive impact on hospital policies and health team. Most importantly training and motivation of health care providers was vital in empowering and supporting mothers to EBF
**Navarro et al. (2013)**	Mother-child dyad Dominican Republic	Quasi-experimental design	IG: *n* = 193 CG: *n* = 259	Home visits by lay health volunteers and group activities biweekly during pregnancy and monthly postpartum, to support BF and newborn care CG: no intervention	Transformational education	Prevalence of EBF	Frequency of EBF through 6 months of age: 7% in IG compared to 2% in CG. Frequency of predominant BF at 6 months of age: 16% in IG compared to 13% in CG	Community interventions lead by lay health volunteers may be a good approach in increasing EBF practices
**Estalella et al. (2020)**	Mothers with late-preterm infants Basque Country, Spain	Quasi-experimental study	*N* = 414 IG: *n* = 161 CG: *n* = 212	IG: postnatal booklet with evidence based on diary feed chart information given at hospital admission CG: standard information given in printed sheet at hospital admission	Not reported	EBF rates at discharge	CG 51% EBF and 38% BF compared to 69% EBF and 26% BF in IG at discharge	Evidence-based BF education designed for this population has been shown to be effective. Communication and shared decision between the health care providers and parents should be practiced improving mothers BF practices.
**Alvarado et al. (1996)**	Pregnant women of low socio-economical level Santiago, Chile	Prospective cohort study	*N* = 128 IG: *n* = 62 CG: *n* = 66	IG: 3 home visits during the last trimester of pregnancy, and one visit in the hospital immediately postpartum by community health promoters; plus, BF education and counseling at health center, 3 times in the first month, and then monthly for the first 6 months postpartum. CG: regular health checkups, with no priority to EBF	Not reported	EBF/BF rates	At 6 months, 42% EBF in IG compared to 0% in CG. BF was practiced in 98% at 6 months	This program with community involvement was significantly effective in promoting EBF. Fundamental factors of a successful BF-promotion program include the positive attitude of the health care team. Home visits produce confidence and commitment to mother, which lead to increased BF
**Langer et al. (1998)**	Primiparous women Mexico City, Mexico	Randomized clinical trial	*N* = 724 CG: *n* = 363 IG: *n* = 361	IG: Doula assisted during labor, childbirth, postpartum, and home visits 40 days after birth to promote early BF to mother and educate on the benefits of BF CG: routine care	Not reported	EBF practices and duration at 1 month postpartum	Frequency of EBF at 1 month was 12% in IG compared to 7% in CG	Psychological support from doulas may have a positive effect on mothers’ knowledge and BF practices.
**Santamaria-Martin et al. (2022)**	Mother-child pair at 10 health centers Madrid, Spain	Randomized clinical trial	*N* = 391 IG: *n* = 206 CG: *n* = 185	IG: PROLACT intervention - 6 weekly BF workshops of 120 mins/each CG: received advice regarding the promotion of BF and benefit of EBF in individual consultations	Not reported	EBF rate and duration at 6 months	At 6-month follow-up: 22% of IG maintained EBF compared to 9% of CG 5% of IG maintained predominant BF compared to 1% of CG Mothers’ adherence and satisfaction with educational intervention were high	Effectiveness of the PROLACT intervention in primary care should be considered evidenced-based practice for implementation in standard practice
**Morrow et al. (1999)**	Pregnant women San Pedro Martir, Mexico	Randomized controlled study	*N* = 125 6 visit IG: *n* = 44 3 visit IG: *n* = 52 CG: *n* = 35	Two IG with different counseling frequencies; 6 or 3 home visits from peer counselor, focusing on various topics on EBF—key family members were included in counseling CG: no intervention	Not reported	EBF rate satisfaction of the mothers with intervention	6 visit group: 80% of participants were EBF at 2 weeks and 67% were EBF at 3 months 3 visit group: 62% of participants were EBF at 2 weeks and 50% were EBF at 3 months CG: 24% of participants were EBF at 2 weeks and 12% were EBF at 3 months Duration of BF was significantly (*p*=.02) longer in IG than in CG All mothers reported peer counselors were helpful and supportive Mothers reported most important source of infant feeding advice was from peer counselor (66%), followed by physicians (19%)	Early and repeated counseling contact promotes successful BF practices. Peer counselors have been effective at supporting mothers in infant feeding and should be accessible to all mothers as a routine component of primary care.
**Gonzalez-Darias et al. (2020)**	Primiparous women with singleton pregnancy Tenerife, Spain	Randomized controlled study	*N* = 391 IG: *n* = 76 CG: *n* = 78	IG: routine postnatal care and 24-h access to BF education through web-based site and 1:1 online contact with a peer supporter for 6 months postpartum CG: routine postnatal care	Not reported	EBF/BF rates	IG (who contacted peer supporter) - 3^rd^ month: 78% of IG were EBF compared to 56% of CG 6^th^ month: 65% of IG were EBF compared to 44% of CG Duration of BF: 3^rd^ month: 88% of IG were BF compared to 73% of CG 6^th^ month: 79% of IG were BF compared to 57% of CG	Web-based program with personal 1:1 interaction has been shown to be effective in maintain BF as it provides mothers with a safe space to seek encouragement and emotional support
**Ulloa Sabogal et al. (2023)**	First-time-pregnant adolescents Santander, Colombia	Randomized, controlled feasibility study	IG: *n* = 43 CG: *n* = 43	IG: received the nursing classification intervention “BF counseling (5244)”in addition to the usual education provided CG: the usual education, no BF intervention given	Not reported	Rate of EBF and BF knowledge	No significant difference in the rates of EBF between the groups Significant increase in mothers’ BF knowledge level after the intervention	Nursing education programs can increase mothers’ knowledge levels. Such programs in combination of other BF interventions should be implemented at hospitals to increase BF practices and patterns
**Franco-Antonio et al. (2022)**	Postpartum women who started BF in the hour after delivery In two public hospitals Southwestern, Spain	Randomized controlled clinical trial	N: 88 IG: *n* = 44 CG: *n* = 44	IG: received a single BF brief motivational intervention CG: received single BF educational session Reinforcement call 1 month postpartum	Health belief model	Prevalence of EBF/BF	Prevalence of EBF at first, third, and six months in IG: 90%, 83%, and 56%, respectively, compared to CG at the same time interval: 64%, 52%, and 20%, respectively. Median EBF duration during first 6 months was 22 weeks in IG compared with 11 weeks in CG Median BF duration was 26 weeks in IG compared to 16 weeks in CG Self-efficacy of mothers was increased by 6 points in IG compared to CG, there was no significant increase	The success of this intervention was due to the therapeutic approach and focus on exploring mother’s intrinsic motivation while encouraging women’s self-discovery and increasing self-efficacy. Also, mothers experienced an increase in community and family support on which they were able to rely on in case of difficulties.
**Ortiz-Felix et al. (2021)**	Pregnant women with 12 weeks of gestation Northeastern, Mexico	Quasi-experimental intervention design	*N* = 60 IG: *n* = 30 CG: *n* = 30	IG: participated in five group sessions and three individual sessions on feeding practices CG: received routine care	Health promotion model	EBF rates	At 4 months, 12% of IG EBF compared to 23% of CG	Prenatal education had a positive effect on infant nutritional status, and promotion of EBF
**Rodriguez-Garcia et al. (1990)**	Pregnant women Four sites in Mexico—Irapuato, Chihuahua, Cuauhtemoc, and Jalapa Mexico	Randomized clinical trial	IG: Irapuato: *n* = 160 Chihuahua: *n* = 122 Cuauhtemoc: *n* = 148 CG (Jalapa): *n* = 155	Irapuato: individual teaching Chihuahua: group teaching Cuauhtemoc: combined model Jalapa: no education provided Community BF education through mass media	Not reported	BF and EBF prevalence and rates	Prevalence of BF in the four communities before intervention ranged from 65% to 81% after intervention 89% of women in the target communities BF compared to 75% before intervention 70% of women EBF during first month compared to 63% in CG	Keys to developing effective BF promotions include providing accurate information to mothers, appropriate education, training, and follow-up; along with a supportive administrative system.
**Balaguer Martinez et al. (2018)**	Mothers of healthy, full-term newborns who are BF or EBF in 5 primary care centers Barcelona, Spain	Randomized unmasked clinical trial	IG: *n* = 193 CG: *n* = 187	IG: routine follow-up visit plus weekly calls, from the same RN, in the first 2 months and biweekly between 2^nd^ and 6^th^ month CG: routine follow-up visits	Not reported	EBF/BF rates	30% of EBF was observed at 6 months in IG compared to 21% in IG No significant difference noted in BF rates of IG vs CG	An essential element to this intervention was the relationship between mothers and healthcare team in building a bond of trust that would serve as a greater motivating force for BF
**Cohen et al. (1999)**	Mothers of full-term willing to EBF for 6 months San Pedro Sula, Honduras	Prospective observational study followed by randomized control trial	*N* = 119	Intensive lactation guidance plus weekly home visits from trained personnel for 6 months and then monthly to 12 months, promoting BF practices	Not reported	EBF prevalence and duration at 4 and 6 months	Mothers’ ability to EBF until 6 months increased from 87% (at 2 weeks) to 97% (at 12 weeks) Mothers reported reasons for EBF: easier, economical, healthier for infants’ growth	Interventions promoting BF should focus on the entire community, not solely on mothers, as well as addressing BF obstacles that can arise. A focus on the early postnatal period should be high priority as it is the most critical time to reinforce desired BF behaviors.
**Guijarro et al. (2014)**	Mothers with healthy newborns until 6 months old Madrid, Spain	Pilot prospective study, controlled nonrandomized	IG: *n* = 57 CG: *n* = 57	IG: two websites used; a no-fee access website and the “Spontania” platform where patients could request telemedicine sessions between routine well visits, along with monthly support group on BF CG: usual follow-up at the primary care clinic	Not reported	EBF rates	EBF through 6 months of age was 36% in IG compared to 19% in CG	Telemedicine is effective and efficient in improving BF rates and resolving questions on BF
**Chumpitaz Durand et al. (2021)**	Pregnant women Lambayeque, Peru	Descriptive, retrospective, and longitudinal design	*N* = 217 CG: *n* = 113 IG: *n* = 104	CG: traditional education IG: multiple intelligences methodology based on the theory by Howard Gardner	Multiple intelligences theory	Knowledge and practices of EBF	57% of the mothers in IG increased knowledge and 68% practiced EBF at 6 months compared with CG 42% improved knowledge and 43% practiced EBF	Educating nursing professionals is critical in improving competencies in providing BF care to mother
**K. Perez et al. (2018)**	Postpartum women in two rural health centers Santo Domingo and El Ayote, Nicaragua	Prospective cohort study	Preintervention: 23 Postintervention: 50	Received “Essential Care for Every Baby” curriculum, including early initiation of BF ad EBF practices	Not reported	EBF practices at 7, 30, and 60 days after birth	EBF increased following birth during the postintervention period compared to the preintervention period: 7 days: 17%–52% (*p* = .007), 30 days: 4%–49% (*p* = .0005) 60 days: 9%–21% (*p* = .2223)	A key to improvements noted was the supportive supervision due to increased motivation and confidence of healthcare workers. In turn, healthcare workers provided BF education with confidence.
***Health institution level***								
**A. Pérez and Valdés (1991)**	Mother-child pairs Santiago, Chile	Prospective intervention study	N: 735 CG: *n* = 313 IG: *n* = 422	IG: BF Promotion Program (BFPP) CG: usual postpartum and infant feeding routine Follow-up every 30 days until 180 days postpartum	Not reported	BF practices and EBF duration and rates	IG began BF at 2.8 h compared to 6.7 h postpartum EBF at 6 months 32% of CG compared to 67% of IG	BFPP has shown to be a success and effective in promoting BF practices Program produced a positive impact on hospital policies, health team, and the health of women and children
**Perez-Escamilla et al. (1993)**	Healthy mothers who planned to BF in two public hospitals Hermosillo, Sonora state, Mexico	Randomized and quasi-experimental study	*N* = 165 Hospital A: 58 Hospital B: 107 IG: *n* = 53 CG: *n* = 54	Hospital A: newborn remained in nursery room Hospital B: newborn roomed in with mother IG: received individual BF guidance CG: normal hospital routine	Not reported	Duration of BF and EBF	IG group had higher (*p* ≤ .05) EBF rates compared to CG in the short term IG had higher BF rates than Hospital A in the long term	Rooming—in with infant along with BF education, benefited lactation performance
**Primary outcome: Any breastfeeding**								
***Individual level***								
**Seguranyes et al. (2014)**	Women receiving antenatal care Catalonia, Spain	Randomized parallel controlled clinical trial	*N* = 1401 IG: *n* = 683 CG: *n* = 718	IG: standard care plus virtual consult with midwife CG: standard care Follow-up after 6 weeks	Not reported	Prevalence of BF at 6 weeks	Prevalence of BF was similar among both groups (IG 64.5% and CG 65.4%)	Virtual care can be effective for postpartum women to promote BF practices
**Sanchez-Espino et al. (2019)**	Pregnant women at 36 weeks of gestation and senior pediatrician residents Nuevo Leon, Mexico	Educational intervention	*N* = 142 mothers *N* = 36 senior pediatrician residents	Two-step intervention: Educate labor and birthing staff at hospital Educate all pregnant women Educational interventions including skin-to-skin contact and early BF practices Follow-up time of 3 months was performed	Not reported	Early BF practices	77% received early BF due to increased knowledge among hospital personal Average onset of early BF in the first and last months was 49 min and 34 min of life, respectively	Promoting early BF is simple and less costly intervention with positive social and economic impacts that can be implemented in hospital setting
**Martinez-Galiano and Delgado-Rodrígueze (2013)**	Primiparous women Four public hospitals in Southern Spain	Prospective cohort	*N* = 520 IG: *n* = 354	Midwife conducted maternal education classes for 75% of the 354 women who attended educational program Followed up for 3 months after delivery	Not reported	Early BF practices	Education provided by midwives achieved early BF (81%) compared with other health professionals (68%)	Educational program ran by midwives showed more satisfaction with maternal education programs, which improved early initiation of BF
**Soriano-Vidal et al. (2018)**	Expectant mothers Valencia, Spain	Multicenter, observational, prospective study	*N* = 212	8-weekly prenatal education classes provided by midwives, including BF	Not reported	Early BF practices	Significant differences between the pre/post scores in early initiation of BF 60% initial session compared to 74% final session (*p* ≤ 0.001)	Prenatal education classes significantly influenced maternal preferences, increasing the involvement of midwives can exert an influence in BF practices.
**Artieta-Pinedo et al. (2013)**	Primiparas women who were followed up from childbirth to end of first year Bizkaia, Northern Spain	Prospective, observational study	*N* = 614	Three groups, according to how many antenatal educational classes women attended (0, 1–4, or 5 or more) Follow-up calls at 1, 3, 6, and 12 months Antenatal education consisted of 2-h classes once weekly for 8 weeks, covering topics related to BF	Not reported	Rate of BF during first year of life	No significant difference with initiation of BF between the groups Positive relationship between attending educational classes and continuation of BF for only the 1st month	Measures to increase BF continuation rates beyond the first month can include postnatal support including strategies for continuing BF with returning to work.
**Bolanos-Villar et al. (2023)**	Infant mother dyad Sonora, Mexico	Prospective study with nonrandomized sampling	*N* = 735 CG: *n* = 179 IG: *n* = 556	IG: routine verbal educational training by hospital personnel plus receiving one of five infographics promoting BF in different perinatal periods CG: routine educational hospital training by hospital personnel	Not reported	BF practices	Mothers in IG BF more (*p* < .0001) than in the CG 92% IG that received at least one infographic BF compared to 78% of CG Three or five infographics in different periods, promoted BF in 95% of participants	Infographics that contain appropriate sociocultural context provides extra support needed to initiate and maintain BF
**Martinez-Galiano and and Delgado-Rodríguez (2014)**	Primiparous women In 4 hospitals across three provinces of Andalusia, Spain	Prospective cohort multicenter study	*N* = 520 IG: *n* = 357	Maternal education classes, including BF benefits, technique and potential problems—midwife was in charge of 75% of women attending	Not reported	Early BF practices and BF maintenance at 2 months	Early initiation of BF 70% of IG compared to 30% who did not receive intervention. Duration of BF at 2 months was seen in 74% of IG compared to 25% who did not receive intervention	This intervention implied that a maternal education program ran by a midwife achieved better outcome than educational program ran by healthcare professional. Mothers valued and had a higher opinion of the program when it was conducted by midwives than by other health professionals.
**Roda et al. (2002)**	Mothers who decided to BF children Ulldecona, Spain	Intervention study	Preintervention: *n* = 125 Postintervention: *n* = 72	BF support policy initiated: information during 3^rd^ trimester including advantages and techniques of BF plus home visits from midwife for motivation and emotional support plus support at pediatric postnatal visit	Not reported	BF duration	Mean duration of EBF was 28 weeks in the postintervention group compared to 18 weeks in the preintervention group	A key concept to the success of this intervention was pediatricians’ ability to promote BF by listening to mothers concerns. Actions for promotion of BF in the primary care setting are a very important factor in BF maintenance.
**Cebrian et al. (2006)**	Children born in 1995 and 1996 (CG) and from 1998 to 2000 (IG) Valencia, Spain	Retrospective evaluation	CG: *n* = 124 IG: *n* = 216	Distributing and explaining a leaflet, including BF support and advantages and stimulation of mothers’ self-esteem distributed by family physician	Not reported	Initiation and duration of BF	52% of infants started BF preintervention group compared to 56% postintervention (not significant) Duration of BF was 3.5 months in preintervention group compared to 3.8 months in postintervention group	
***Community level***								
**Monterrosa et al. (2013)**	Women with children 6–24 months from 6 low-income communities Morelos, Mexico	Quasi-experimental cluster design	IG: *n* = 266 (Morelos) CG: *n* = 201 (Puebla)	IG: Nurses delivered 5 scripted messages, including BF through radio 7 times each day on 3 radio stations for 21 days CG: no exposure to messages	Theory of planned behavior	BF frequency	In IG, BF frequency was 3.7 times/d higher than in CG	Theory-based nutrition communication strategies using scripted messages have been shown to be effective in improving BF frequencies. This strategy is simple and feasible to produce in low-income communities.
**Gamez Requena et al. (2004)**	Pregnant women who had cesarean section Malaga, Spain	Retrospective study	*N* = 152 IG: *n* = 76 (1996) IG: *n* = 76 (1998)	During 1997, BF-promotion activities were organized, including workshops aimed at healthcare staff, public events to promote BF, and changes in hospital care	Not reported	BF rates and duration	In 1996, only 28% of women started BF compared to 1998 percentage multiplied by 3, reaching 85% (*p* < .0005). Duration of lactation increased from 130 days on average in 1996 to 163 in 1998 (*p* < .215)	Promotion of BF should be integrated as part of the procedures and care protocols in hospitals
**Primary outcome: Breastfeeding knowledge and attitude**								
***Individual level***								
**Arias Ramirez, et al. (2021)**	Pregnant women Villavicencio, Colombia	Quasi-experimental pre- and post-study	*N* = 275	BF educational strategy designed and implemented in four phases Phase 1: application of survey Phase 2: Design and strategy of intervention; including teaching materials on the benefit of BF phase 3: Intervention implemented Phase 4: Impact of strategy implementation Follow-up after 6 months of intervention	Not reported	KAP of pregnant women and nursing mothers for 6 months	KAP increased significantly after 6 months of educational intervention: Knowledge: 99% (*n* = 220) Attitudes: 99% (*n* = 219) Skills: 86% (*n* = 190) BF children up to 2 years of age increased from 17% to 51%	A thorough well-planned educational program based on the cultural beliefs and environment of mothers are fundamental in promoting mothers BF KAS
**Rojas et al. (2019)**	Mothers with children under 2 years of age Caracas, Venezuela	Cross-sectional investigation	*N* = 1,132	Educational sessions were held on multicomponent BF issues and benefits during two 60-minute sessions	Not reported	Knowledge on BF	After educational intervention knowledge increased from 29% to 60%	Teaching educational interventions utilizing appropriate methods shows effectiveness in increasing knowledge of BF practices and can encourage the maintenance of EBF until 6 months of infant’s life.
**Morales et al. (2021)**	Pregnant women during 3^rd^ trimester Granma Province, Cuba	Quasi-experimental study	*N* = 40	Weekly workshops for 6 weeks, with a duration of 40 to 45 min, including perceived benefits of BF Pre and post questionnaire	Not reported	BF knowledge	Before intervention, 62% perceived benefits for EBF; after intervention, 100% had identified the benefits of EBF for 6 months	From early stages of pregnancy, it is important that specific BF knowledge be provided to mothers to ensure awareness of the vital importance of BF. Healthcare professionals also play a fundamental role due to their frequent interaction with mothers, and they can offer valuable information related to BF practices.
**Leyva et al. (2015)**	Mothers with children under 6 months of age Trujillo, Peru	Quantitative, pre-experimental with pre-post evaluation, no control	*N* = 55	Educational program based on the Cross-Cultural Nursing Theory of Madeleine Leininger	Not reported	BF knowledge and beliefs	Before intervention: 96% of mothers obtained average level in BF knowledge and 4% showed high knowledge After intervention: 100% achieved high level of knowledge about BF (*p* = .000)	The level of knowledge that the mother has about BF is what encourages the mother to be responsible and ensure lactation effectively to their children.
**Tamayo Velázquez et al. (2022)**	Postpartum women Gibara Municipality, eastern Cuba	Quasi-experimental study	*N* = 35	Six-weekly educational intervention preparing mothers in BF techniques and benefits	Not reported	Knowledge on BF 6 months follow-up	Before intervention, knowledge was adequate in 6 patients (17%); after intervention, 33 people (94%) had adequate knowledge	Face-to-face communication is the best method that best allows the incorporation of cultural factors. It is encouraged that health professional encourage at every encounter with mother the benefits and details of BF.
***Health institution level***								
**Valdes et al. (1995)**	Health professionals Santiago, Chile	Quasi-experimental Pre-post study	*N* = 360 at baseline and 318 postintervention	3-Day multicomponent training program including BF management and knowledge on clinic support for BF women	Not reported	Knowledge of health care providers regarding BF support and promotion	28% increase in teaching BF techniques to pregnant women 86% recommend EBF for the optimal period of 6 months Health center showed 30% increase BF practice	Well-organized and intensive lactation course can have a major impact on the clinical practices in hospital staff, these practices are extremely important for BF promotion
**Lasarte Velillas et al. (2007)**	Pediatric residents from four hospitals Madrid, Tenerife, Tarragona and Zaragoza, Spain	Quasi-experimental study (pre-post test)	*N* = 42 88% women 12% men	Received monthly intensive theoretical BF training after took weekly turns answering parents’ questions in a forum Pre-post knowledge test given	Not reported	Knowledge and skills on BF	Rate of correct answered increased from 80% to 88% posttest/intervention Residents stated that nearly half (44%) of patients needed support with BF	Training future pediatricians on BF management has been shown to contribute to the improvement of BF knowledge
**Iñarritu-Pérez et al. (2022)**	Undergraduate medical students Mexico	Quasi-experimental study (pre-post test)	*N* = 154 Females: 67% Males: 32%	Evidenced-based workshop covering various topics and activities related to BF	Not reported	Knowledge and attitude about BF	Increase in knowledge: Females: 30%–95% Male: 26%–84% Positive attitudes: Females: 30%–91% Males: 34%–82%	Educational interventions for healthcare professionals influence the increase in women’s BF practices
**Velásquez Rondón and Huaman Hernández (2020)**	Primigravid mothers Arequipa, Peru	Quasi-experimental study (pre-posttest)	*N* = 30	Nursing Care intervention based on Kristen Swanson’s theory	Not reported	Knowledge and acceptance of BF practices	Before intervention: 63% regular EBF knowledge level and 37% poor EBF knowledge level After intervention: 73% with good EBF knowledge and 27% regular EBF knowledge level	Nursing staff need an adequate and solid training in BF, since healthcare professionals play an important role in improving the expected outcomes of mothers’ EBF
***Community level***								
**Prieto et al. (2017)**	Mothers San Juan Sacatepequez, Guatemala	Pilot impact evaluation and qualitative study	IG (I) *n* = 24 IG (II) *n* = 32 IG (III) *n* = 30 CG: *n* = 14	IG (I): received health promoting text messages twice a week, including BF IG (II): peer-to-peer groups formed IG (III): peer-to-peer groups formed and participation of healthcare professional in group discussion plus participants received health promoting texts, including BF IG (IV): given a mobile phone to be used for matters related to infant, no further intervention CG: simply given a cell phone	Not reported	BF Knowledge and self-reported behavior 23-week intervention	Most effective intervention in terms of improved levels of knowledge is through direct one-way communication delivering EBF messages (IG I). Followed by peer-to-peer groups with a medical professional in the group (IG III).	BF promoting text messages can raise awareness among mothers in countries where health service resources are limited. Health professionals play an important role in providing reliable health information and counteracting inaccurate recommendations to
**Hernández Perez et al. (2018)**	Students, 3^rd^ and 4^th^ year of High School Tenerife, Spain	Longitudinal study of education intervention (pre-post) and randomized control trial	*N*= 970 IG: *n* = 525 CG: *n* = 445	Both groups completed pre and post CG: no intervention IG: talk, video projection, informative leaflet, narrative short stories and role-play activities related to BF	Not reported	Knowledge and attitude toward BF	Postintervention showed increase in level of knowledge from 71% in IG compared to 54% in CG	Decision to BF begins before pregnancy, educating adolescent students of both sexes is effective in improving knowledge and attitudes toward BF

*Note.* BF = breastfeeding; CG = control group; EBF = exclusive breastfeeding; IG = interventional group; KAP = knowledge, attitude, and practice.

Most of the studies reported positive changes in knowledge, attitudes, and/or practices of breastfeeding. There were various forms of interventions used ranging from participants receiving a single brief motivational session (Gámez Requena et al., 2004) to intensive lactation education plus support from trained personnel through weekly and monthly home visits for 1 year (Estalella et al., 2020). Many of the interventions included patients receiving educational sessions, eight included home visits from trained personnel (Alvarado et al., 1996; Burkhalter & Marin, 1991; Cohen et al., 1999; Langer et al., 1998; Lucchini Raies et al., 2013; Morrow et al., 1999; Navarro et al., 2013; Roda et al., 2002), and one receiving support from a doula (Langer et al., 1998). Four studies reported on peer support (Burkhalter & Marin, 1991; Gonzalez-Darias et al., 2020; Morrow et al., 1999; Prieto et al., 2017), and six studies utilized video, radio, phone, virtual, or telehealth interventions (Guijarro et al., 2014; Hernández Perez et al., 2018; Maslowsky et al., 2016; Monterrosa et al., 2013; Prieto et al., 2017; Seguranyes et al., 2014). Three studies noted no significant difference between control and intervention groups (Artieta-Pinedo et al., 2013; Balaguer Martínez et al., 2018; Ulloa Sabogal et al., 2023).

### Outcome: Exclusive Breastfeeding

Across all studies, exclusive breastfeeding rates either increased or were maintained. For example, a breastfeeding program that included community health promoters during prenatal and postnatal home visits for 6 months in Chile reported that exclusive breastfeeding at 6 months was significantly higher in the intervention group than in the control group (42%; 0%, *p* ≤ .01; Alvarado et al., 1996). However, two interventions showed no significant difference in intervention group compared to control group, which included home visits from lay community volunteers (Navarro et al., 2013) and home visits from a doula (Langer et al., 1998). A nursing educational intervention in a hospital in Colombia showed no significant difference in the rates of exclusive breastfeeding at 6 months between groups; however, significant improvements in knowledge of breastfeeding were reported (Ulloa Sabogal et al., 2023).

Nine randomized control trials in this review had significantly higher rates of exclusively breastfeeding in the intervention group than in the control group (Balaguer Martínez et al., 2018; Bueno-Gutiérrez et al., 2021; Cohen et al., 1999; Franco-Antonio et al., 2022; Langer et al., 1998; Lucchini Raies et al., 2013; Rodriguez-Garcia et al., 1990; Santamaría-Martín et al., 2022; Seguranyes et al., 2014). A telephone-delivered educational session in Ecuador, given by trained breastfeeding nurses, and access to on-call nurse for 30 days reported significantly higher rates of exclusively breastfeeding in the intervention group than in the control group at 3 months; 87% compared to 67% (Maslowsky et al., 2016). A prenatal breastfeeding skills group in Chile, which included training of the health care team and implementation of breastfeeding education and hands-on skills activities to initiate and maintain breastfeeding, reported 67% of mothers in the intervention group were exclusively breastfeeding at 6 months compared to 32% of the control group (A. Pérez & Valdés, 1991). A multicomponent prenatal and postnatal educational program in Chile, using lectures, monthly support groups, peer support, and home visits, reported 74% of intervention group exclusively breastfeeding compared to 30% in the control group, at 6 months (Burkhalter & Marin, 1991). In a hospital in Mexico, mothers who planned to breastfeed delivered in a maternity ward where either the newborn remained separated in a nursery room or the newborn roomed in with mother. Mothers who roomed in with newborns either received individual breastfeeding guidance during the hospital stay or received the hospital’s usual support. This intervention reported that mothers who roomed with newborns and received breastfeeding guidance showed a significant higher rate of exclusively breastfeeding throughout the first 4 months compared to the infants who went to the nursery (*p* < .05; Perez-Escamilla et al., 1993). Mothers with late pre-term infants in Spain received a postnatal booklet with evidence-based easy-to-understand breastfeeding education along with a feeding chart diary, and results showed 68% of infants in the intervention group were exclusively breastfeeding compared to 51% in the control group at discharge (Estalella et al., 2020).

The breastfeeding promotion programs in Chile, a health system–based intervention including training of health care team in breastfeeding along with prenatal educational activities and open outpatient lactation clinic for mothers, produced a positive impact on exclusively breastfeeding at 6 months in the intervention group compared to the control group (Pugin et al., 1996; Valdés et al., 1993).

### Outcome: Any Type of Breastfeeding

In a quasi-experimental media intervention in Mexico, nurses delivered breastfeed promotion through scripted messages via radio, for 21 days; the intervention group reported a 3.7-times higher frequency of breastfeeding than the control group which did not receive any messages (Monterrosa et al., 2013). In Spain, a community intervention organized activities to promote breastfeeding such as workshops aimed at health care professionals, hosted public events, and implemented changes in hospital health care services, and reported results showed that 85% of women in the intervention group started breastfeeding compared to 28% of women in the control group (Gámez Requena et al., 2004). In a prospective study, in Mexico, mothers were provided with printed infographic materials, at different perinatal periods, promoting breastfeeding, and results showed that 92% of mothers in the intervention group who received three or more printed infographics planned to breastfeed compared to 78% of the control group (Bolaños-Villar et al., 2023). A two-step educational intervention carried out in a public hospital in Mexico provided breastfeeding training for the labor and delivery staff of the hospital and educated participants on early breastfeeding; results showed early breastfeeding were achieved in 77% of the cases (Sanchez-Espino et al., 2019). One intervention in Spain relating to an educational intervention involving virtual consultations from a midwife reported no significant difference of breastfeeding prevalence between intervention and control groups: 64.5% and 65.4% (Seguranyes et al., 2014); however, in another study, an educational intervention conducted by a midwife achieved early breastfeeding (81%) compared with educational intervention given by other health care professionals (68%; Martínez-Galiano & Delgado-Rodríguez, 2014). An educational intervention for primiparous women showed no significant difference with initiation of breastfeeding between control and intervention groups; however, a positive relationship was reported between attending educational classes and continuation of breastfeeding for the first month only (Artieta-Pinedo et al., 2013).

### Outcome: Knowledge and Attitudes

The level of knowledge a mother has regarding the importance of breastfeeding is one of the major factors that influence breastfeeding duration and abandonment (Ávila-Ortiz et al., 2020; Vázquez-Osorio et al., 2022). Results from studies identified in this review showed that educational interventions significantly improved mothers’ level of knowledge about the importance of exclusively breastfeeding for 6 months. For example, one study reported improved results after the intervention based on multiple intelligence, 57% of the mothers developed more knowledge than 42% of mothers who received traditional education (Chumpitaz Durand et al., 2021). Similar results were reported in another study (Rojas et al., 2019), where 29% of mothers with deficient level of knowledge improved to 60% with good level of knowledge among 1,132 pregnant women and mothers who attended educational breastfeeding workshops. Another study (Leyva et al., 2015) reported that after applying an educational program to a group of 55 mothers of children younger than 6 months from a health center in Trujillo, Peru, they managed to change the level of knowledge with respect to breastfeeding, from the average level of 96% to 100% at high level. In Cuba, weekly workshops for pregnant women during their third trimester resulted in a 100% perceived benefit for breastfeeding for 6 months compared to 62% before intervention (Morales et al., 2021). With regards to the effectiveness of mothers understanding the benefits of breastfeeding infants up to 2 years of age, percentages ranged from 18% before intervention to 51% after intervention in a study in Colombia (Arias Ramírez et al., 2021). A cross-sectional investigational study of mothers in Venezuela reported that topics with the greatest impact included milk conservation, breastfeeding practices, and measures to increase milk production and that mothers’ knowledge level increased from 29% to 61% (Rojas et al., 2019).

Three quasi-experimental (pre-posttest) studies on health care professionals receiving multicomponent intensive training and educational workshops, including about breastfeeding, showed that the percentage of correct answers increased from 80% pretest to 88% posttest (*p* = .0028; Lasarte Velillas et al., 2007), and knowledge level increased from 30% to 95% in female medical students and 26% to 84% in medical students, along with an increase in positive attitude toward breastfeeding after intervention; 91% in female medical students and 82% male medical students (Iñarritu-Pérez et al., 2022). A study in Guatemala reported that the most effective intervention in terms of improved levels of knowledge was through one-way communication, utilizing breastfeeding promotional texts (Prieto et al., 2017). A two-phase program conducted in the Dominican Republic with adolescent mothers aimed to discuss their postpartum experience, and an interventional program was created based on the topics that the adolescents felt were the most important to them. Participants indicated that the highest priority of desired knowledge included maternal lactation and infant feeding (Navarro et al., 2013). These results show that programs designed to improve knowledge and attitudes about breastfeeding are feasible and should be offered as part of a routine primary care component.

## Discussion

The objective of this scoping review was to examine the existing available literature on interventions that promote breastfeeding among pregnant women/mothers residing in SSSCs. These interventions showed a positive impact in promoting breastfeeding knowledge, attitude, and practices of breastfeeding, exclusive breastfeeding, early initiation, and duration of breastfeeding, including knowledge and confidence of health care professionals. Similar results were found in a systematic review (Sinha et al., 2015) which reported that educational interventions and counseling have the greatest impact on promoting maternal breastfeeding, including exclusive breastfeeding for 6 months of infants’ age. These findings show that there is an urgent need to increase and invest in continued support for breastfeeding in SSSCs, through implementation of education and training to individuals, community, and health care system.

Successful interventions included in this review were provided by trained health care professionals, peer counselors, or lay community volunteers, as well as from doulas and midwives. A key success to these interventions was that mothers were drawn to the emphatic, reassuring, and motivating support from the trained health care professionals and peer counselors. The relationship between mothers and her health care team is important in building and maintaining a bond of trust that would serve as a greater motivating factor for breastfeeding. Breastfeeding counseling should be provided as a continuum of care, by appropriately trained health care professionals and community-based lay and peer breastfeeding counselors who are associated with positive attitudes and more confidence when providing breastfeeding recommendations and support to mothers.

This review showed that the increase in practice of exclusively breastfeeding up to 6 months resulted from education and counseling interventions. The studies in this review showed significantly higher rates of exclusive breastfeeding are observed when educational group discussions are used as the principal strategy, and the majority occurred in the postnatal period. It has been noted that a focus on the early postnatal period should be of high priority as it is the most critical time to reinforce desired breastfeeding behaviors. Successful programs provided education and information about breastfeeding to pregnant women and mothers during the prenatal and postnatal periods and included training of health care team and lactation support groups for mothers who were nursing. Such programs should provide timely and repeated breastfeeding interventions, including education of health care staff, and it provided the necessary support to help mothers successfully achieve breastfeeding. Future studies could include longer duration of intervention to determine if countries are reaching the WHO global target of 50% prevalence of exclusive breastfeeding by 2025.

A gap found in the scoping review was lack of familial intervention, that is, involving fathers or family members, and so on, as many mothers report needing more support from significant family members throughout childbirth and postpartum period. In other breastfeeding education and intervention studies, familial support was found to significantly and positively predict changes in knowledge and attitudes related to breastfeeding and to increase the practices of breastfeeding including incidence and duration (Ekström et al., 2003; Hanafi et al., 2014; Haneuse et al., 2000; Ratnasari et al., 2017; Swanson & Power, 2005), suggesting that interventions that include components of education for family members in support of breastfeeding may increase their intended effect. A lack of support from partners and family serves as an obstacle to mothers achieving their breastfeeding goals and in turn leads to reduced rates of breastfeeding. There is a strong need for public health messages not solely targeted toward mothers but also with a focus on their significant others and families which would be vital when developing breastfeeding campaigns. Indeed, a focus group found that among Mexican-American mothers in the United States, a lack of partner and family support was found to be a barrier to breastfeeding, even when knowledge about the benefits of breastfeeding was high (Gill et al., 2004). Understanding such barriers to breastfeeding can suggest areas for curricular and intervention improvement in support of increased incidence and duration of breastfeeding. More research that examines the crucial role that significant others and family play in promoting and supporting breastfeeding is warranted.

Along with family support, cultural beliefs and practices are also very important components in promoting breastfeeding. It has been found in other studies cultural tailoring or incorporation of different cultural, traditional, and/or familial values can positively impact the efficacy of breastfeeding interventions (Cidro et al., 2015; Cook et al., 2021; MacVicar et al., 2015; Rhodes et al., 2021; Segura-Pérez et al., 2021). Interventions that utilized cultural tailoring or peer support for Hispanic women were found to increase exclusive breast feeding and the duration of breastfeeding (Linares et al., 2019; Lutenbacher et al., 2018). In general, it has been found that cultural tailoring of health promotion interventions tends to increase the efficacy of the initiative (Chandler et al., 2022; Ehrlich et al., 2016; McCurley et al., 2017). Because the rates of breastfeeding are low across Spanish-speaking countries, it is imperative to understand how to design the most impactful interventions to increase breastfeeding incidence and duration.

Another observation found in this review was that the majority of the studies took place in either Spain or Mexico. No study was identified during the literature search that was conducted in Argentina, Bolivia, Costa Rica, El Salvador, Panama, Paraguay, Uruguay, and the Spanish-speaking central African country of Equatorial Guinea. Consequently, more studies including those conducted in these countries are needed to add to the literature on the effectiveness of breastfeeding interventions in these identified SSSCs. Understanding cultural practices from different countries across SSSCs can offer a wider explanation to the variation in successful promotions that can increase breastfeeding practices, knowledge, and attitudes across different countries.

To help meet the WHO recommendations of continued breastfeeding along with introducing appropriate complementary foods for up to 2 years of age or longer, breastfeeding interventions should be given at both antenatal period and postnatally for up to 2 years. No study in this scoping review analyzed the effects of breastfeeding up to 2 years of age or beyond. Further research is needed regarding promotional interventions within Spanish-speaking countries in maintaining breastfeeding practices for more than 24 months. A systematic review and meta-analysis found that of the articles included and assessed as “continued breastfeeding” (12–23 months in duration), the interventions evaluated showed a significant improvement of 61%, with higher effects in higher-income and urban settings than in low-moderate-income and rural settings (Sinha et al., 2015). However, studies that include either intervention duration of or follow-up at 2 years are incredibly limited and thus offer additional opportunities for research.

Scaling up breastfeeding policies and programs effectively has been identified as a global health priority for its role in achieving the Sustainable Development Goals. There was a lack of policy-related interventions in this scoping review. Poor regulation of marketing campaigns for artificial foods and the lack of funding for programs that promote, protect, and support breastfeeding are barriers in SSSCs that national policies could address.

Mobile health strategies could also serve as a potential vehicle to promote breastfeeding and improve levels of knowledge through direct communication delivering breastfeeding messages. Text messaging programs could be informative and improve exclusive breastfeeding rates and duration in communities with low prevalence of breastfeeding practices. Mobile health applications are becoming increasingly popular in SSSCs (Farach et al., 2015), which can benefit communities by making breastfeeding promotion more accessible and affordable. Further research is needed to test how best they can be used to promote breastfeeding.

### Implications

Interventions in this review demonstrate a positive relation between breastfeeding interventions and knowledge, attitude, and practices. These findings were consistent with previous systematic reviews conducted with nurses, midwives, and physicians, which reported increased knowledge and breastfeeding skills and improved attitudes toward breastfeeding following their participation in educational programs (Sandhi et al., 2023). The implications of these findings are summarized in Table 4 and expanded below.

**Table 4:** Implications for Practice, Policy, and Research

Practice • Even basic interventions such as providing educational handouts showed some efficacy at increasing breastfeeding knowledge and practice • Multiple modalities, including low-cost phone counseling sessions and radio messaging are effective in increasing breastfeeding knowledge and practice • The most effective interventions at increasing breastfeeding adherence and duration included antenatal and postnatal education and support • Increasing the knowledge of healthcare professionals on the benefits of breastfeeding increased their patients’ adherence to breastfeeding • In most of the studies assessing knowledge, increasing the knowledge of mothers regarding the importance of breast feeding increased the act of breastfeeding
Policy • Increase financial support for development of education and interventions promoting breastfeeding • Provide educational and training opportunities for healthcare professionals that work with women in both antenatal and postnatal settings • Develop or invest more in support structures for non-hospital healthcare workers such as doulas, peer breast feeding counselors, and community workers that interact with pregnant and postpartum women • Increase public health messaging to bring visibility to and understanding of the health benefits of breastfeeding for infants and mothers • Regulate marketing to increase support for breastfeeding
Research • More rigorous research on interventions that are longer in duration • Investigations into effects of familial and postnatal support interventions (i.e., education for fathers, extended family caregivers, employers, community health services) • Investigate the impact on breastfeeding duration through community-based and environmental interventions • Address the research gap found in a lack of breastfeeding education interventions and services in Argentina, Bolivia, Costa Rica, El Salvador, Panama, Paraguay, Uruguay, and the Spanish-speaking central African country of Equatorial Guinea • Investigate the impact of culturally-tailoring breastfeeding interventions specific to each country/region • Investigate mhealth (mobile-health delivery) options as another low-cost way to increase knowledge of and adherence to breastfeeding in SSSCs

Initiatives that include giving mothers the support they need to breastfeed, programs that educate and provide support to mothers, as well as education and training in breastfeeding for all health professionals that provide health care for mothers and children are highly effective. Yet the most effective intervention to improve and maximize breastfeeding practices includes promotional activities, educations workshop, and training of health care staff along with changes of policies in health care settings. The influence of maternal education is critical at increasing breastfeeding practices and durations in SSSCs.

## Conclusions

Breastfeeding is a highly cost-effective, disease-preventive intervention with a global health priority. This review suggests that breastfeeding education should be offered as part of a routine primary care component. This current review has found that even low-cost approaches to increasing knowledge of the benefits of breastfeeding among health care providers, in mothers prepartum and postpartum, and in a variety of settings have the ability to increase both knowledge of and adherence to breastfeeding. In addition, a supportive environment that includes health care professionals in both the hospital and community environments, as well as peer and community support, can impact the incidence of breastfeeding. Ensuring that messaging and education is culturally tailored has the potential to increase efficacy of breastfeeding education interventions and initiatives. Future research is needed on the impact of promoting an environment in which breastfeeding is encouraged and supported through larger public health policy initiatives and is important in creating universal support for breastfeeding mothers. This could include targeted marketing campaigns, education for extended family (such as fathers and other caregivers) and community members (such as the important role employers can play in supporting working mothers that have to pump and store breastmilk through options such as breast pumping rooms and dedicated breast milk storage), and state-sponsored education on the public health importance of breastfeeding in the media. Appropriate global policies and programs, providing breastfeeding education and counseling, as well as training health care professional should be implemented to create a positive environment for successful breastfeeding in SSSCs.

## Limitations

There are some limitations to this scoping review that are worth noting. Although the authors searched several biomedical databases using various search terms, relevant intervention studies may have been missed. This review did not differentiate how many references were ascertained from each of the searched databases. Also, scoping reviews do not necessarily employ certain methodologically solid methods such as quality assessment and bias risk appraisals, as in the case of systematic reviews.
